# Who Eats Whom in a Pool? A Comparative Study of Prey Selectivity by Predatory Aquatic Insects

**DOI:** 10.1371/journal.pone.0037741

**Published:** 2012-06-05

**Authors:** Jan Klecka, David S. Boukal

**Affiliations:** 1 Department of Ecosystems Biology, Faculty of Science, University of South Bohemia, České Budějovice, Czech Republic; 2 Laboratory of Theoretical Ecology, Biology Centre of the Academy of Sciences of the Czech Republic, v.v.i, Institute of Entomology, České Budějovice, Czech Republic; Northwestern University, United States of America

## Abstract

Predatory aquatic insects are a diverse group comprising top predators in small fishless water bodies. Knowledge of their diet composition is fragmentary, which hinders the understanding of mechanisms maintaining their high local diversity and of their impacts on local food web structure and dynamics. We conducted multiple-choice predation experiments using nine common species of predatory aquatic insects, including adult and larval Coleoptera, adult Heteroptera and larval Odonata, and complemented them with literature survey of similar experiments. All predators in our experiments fed selectively on the seven prey species offered, and vulnerability to predation varied strongly between the prey. The predators most often preferred dipteran larvae; previous studies further reported preferences for cladocerans. Diet overlaps between all predator pairs and predator overlaps between all prey pairs were non-zero. Modularity analysis separated all primarily nectonic predator and prey species from two groups of large and small benthic predators and their prey. These results, together with limited evidence from the literature, suggest a highly interconnected food web with several modules, in which similarly sized predators from the same microhabitat are likely to compete strongly for resources in the field (observed Pianka’s diet overlap indices >0.85). Our experiments further imply that ontogenetic diet shifts are common in predatory aquatic insects, although we observed higher diet overlaps than previously reported. Hence, individuals may or may not shift between food web modules during ontogeny.

## Introduction

Who eats whom and how much? Answering this seemingly simple question is vital for the understanding of processes structuring animal communities. Data on prey selectivity are crucial for mapping the topology of food webs and predicting the effects of species invasions and extinctions on food web structure and stability [1]–[4]. Data on the diets of different predators are also required to quantify resource partitioning, which can underlie their coexistence [5], [6]. Yet for many food webs, publication of detailed data on the trophic links is sacrificed to achieve more compact description of the often complex food web topology [3], and data coverage varies across habitat types. In freshwater, food webs in standing fishless water bodies have been much less studied than those in streams and lakes (see [7]). Different physical factors and biotic interactions shape the communities in these habitat types, and many species are present in only one of them [8]. Conclusions drawn from the studies of food webs in streams and lakes may thus have only limited applicability to small standing waters without fish. For example, predator-prey body mass ratios differ across habitat types and taxonomic groups of consumers, which may have important implications for food web stability because predator-prey body mass ratios affect interaction strengths [9], [10].

Higher trophic levels in small standing waters are occupied by anurans and aquatic insects (e.g., [11]). The top predators include, at least in the temperate zone, mainly dragonfly and damselfly larvae (Odonata), diving beetles (Coleoptera: Dytiscidae) and bugs (Heteroptera: Nepomorpha). All three groups are speciose and diversified [12] and tens of species can coexist locally (e.g., [11], [13]). They have been traditionally considered as generalist predators [14]–[16], most likely because of the paucity of experimental data. However, many empirical studies suggest that these predators frequently prefer some prey over others (e.g., [17]–[20]). Their prey selectivity may lead to cascading effects in the food web [21] and contribute to the maintenance of high levels of biodiversity in standing waters.

Although predatory aquatic insects have been studied for decades, their feeding relationships are surprisingly little known apart from a few model taxa. A synthesis of their prey selectivity is missing and available data need to be described in detail. Hence, it cannot be assessed to what extent the mechanisms of selective predation and resource/habitat niche partitioning promote the diversity of communities in small water bodies, e.g. through food web compartmentalization [22], [23]. Neither do we know which predators have the largest impact on food web structure and which prey are keystone species supporting a disproportionate number of predators in these habitats.

To help answer these questions, we carried out a series of multiple-choice predation experiments with common predator and prey species that often coexist in pools and other small standing water bodies in central Europe. Experiments are the only viable option to compare the diets and prey selectivity across all these predators, as bugs and diving beetle larvae are suctorial. Gut content analyses based on morphological identification of the remains in the gut of the predator are hence applicable only to dragonfly larvae [24], [25] and adult diving beetles. Even when gut contents can be analysed, the estimates of prey selectivity and consumption rates may be severely biased by the fact that different types of food may take very different times to pass through the gut (e.g., [26]). Moreover, neither stable isotope analysis [27]–[29] nor gut contents can reveal predator selectivity in the absence of detailed data on available prey [18].

In this paper, we summarize our experimental results on selective predation by diving beetles, bugs and odonate larvae together with previously published experiments. We subsequently discuss the importance of diet overlaps, varying vulnerability of prey and ontogenetic diet shifts for the structuring of food webs in small fishless water bodies. The influence of body size and other trophic traits on the strength of predation links will be thoroughly analysed elsewhere (Klecka & Boukal, in prep.).

## Methods

### Ethics Statement

No specific permits were required for fieldwork as the sampled localities are not protected or privately owned. The use of tadpoles in the experiment was permitted by the regional authority (permit no. KUJCK 12524/2010 OZZL/2/Do) and the Ministry of Education of the Czech Republic (permit no. 7947/2010 30). No permit was needed for the use of invertebrates in the experiment because none of the species is protected.

### Laboratory Experiment

We performed multiple choice predation experiments in an experimentally assembled, semi-natural food web with nine regionally common species (13 different stages) of predatory aquatic insects (Table 1) and seven prey species (Table 2). We also used different stages of three predators to study ontogenetic diet shifts. We were not able to cover more species or stages due to limited time available for the experiment, constrained chiefly by the availability of small tadpoles. *Acilius* and *Libellula* were chosen because they were among the most abundant species in the field and multiple stages were available simultaneously during the experiment. *Dytiscus* was used because both larvae and adults are voracious predators [30]–[32] that may even cause trophic cascades [21]; understanding the differences in their diets could help assess their potentially contrasting impacts on prey populations.

**Table 1:** Predators used in the experiments.

Species	*N*	Body length (mm)		Foraging microhabitat
		Mean	SD	
**Coleoptera: Dytiscidae**				
*Hydaticus seminiger* (A)	8	14.8	0.29	bottom
*Acilius canaliculatus* (L2)	7	12.9	0.54	water column
*Acilius canaliculatus* (L3)	8	21.7	1.90	water column
*Acilius canaliculatus* (A)	8	15.4	0.70	bottom
*Dytiscus marginalis* (L3)	5	47.8	2.95	bottom
*Dytiscus marginalis* (A)	9	32.9	0.81	bottom
**Heteroptera: Nepomorpha**				
*Ilyocoris cimicoides* (A)	8	14.1	0.54	bottom
*Notonecta glauca* (A)	8	15.1	0.42	water column
**Odonata**				
*Coenagrion puella* (F-0)	9	12.5	0.90	water column^a^
*Libellula depressa* (F-2)	7	15.3	0.63	bottom
*Libellula depressa* (F-0)	6	21.9	1.18	bottom
*Sympetrum sanguineum* (F-0)	8	15.7	0.80	bottom
*Anax imperator* (F-0)	9	48.1	2.54	bottom

a spent most time on the perching sites.

Foraging microhabitat: predators crawling on supporting plastic mesh classified as foraging in water column. Stage given in parentheses: A = adult, Ln = larva of n-th instar; F-n = larva of n-th instar before the last. *N* = number of replicates (individual predators).

**Table 2:** Prey species used in the experiments.

Species	Body length (mm)		Microhabitat occupation	Mortality in control trials (%)	Taxon (order)
	Mean	SD			
*Asellus aquaticus* (A)	7.63	1.03	bottom	0.0	Isopoda
*Chironomus* sp. (L)	9.38	0.64	bottom	11.7	Diptera
*Cloeon dipterum* (L)	6.51	0.73	bottom	3.3	Ephemeroptera
*Culex* sp. (L)	9.16	0.34	water column	3.3	Diptera
*Daphnia* sp. (A)	2.34	0.22	water column	6.1	Cladocera
*Lymnaea stagnalis* (L) - shell	13.19	1.87	water column^a^	0.0	Pulmonata
*Rana arvalis* (L) - SVL	6.33	0.30	bottom	0.0	Anura
*Rana arvalis* (L) - TL	19.33	0.92			

a spent most time crawling on the sides of the experimental vessel.

Stage given in parentheses: A = adult, L = larva. SVL = snout-vent length, TL = total length.

Experiments were carried out in May and June 2010 in a climate room with a regular temperature cycle (day: max. 22°C, night: min. 18°C) and 18 L:6D photoperiod. All animals were collected at various sites in South Bohemia (Czech Republic) and acclimated for 2–5 days prior to experiments. Predators were kept individually in small containers (0.25–0.7 l) and fed daily ad libitum with prey different from those used in the experiments (mainly larvae of Trichoptera). Each predator was starved for 24 hours prior to the experiment to standardize its hunger level. Prey were kept in larger containers (2–20 l) and supplied with abundant natural food (decaying plant material, detritus, algae etc.). Prey individuals which were unused or survived the experiment were released to their natural habitat.

Experiments were performed in translucent whitish plastic boxes (bottom dimensions 24×16 cm) filled with 2.5 litres of tap water (depth ca. 8 cm) aged for one day. The vessels had no substrate on the bottom; four narrow stripes of white plastic mesh suspended vertically in the water column provided simple perching sites. In each replicate, all prey individuals were released first (six *Rana* tadpoles, six *Lymnaea*, 10 *Chironomus*, 10 *Cloeon*, 10 *Culex*, 10 *Asellus* and 30 *Daphnia*; the densities were within the range of natural densities observed in small pools in the field); the predator was added after several minutes. Each experiment was left undisturbed to run for 24 hours, after which we counted all surviving prey; hence, dead prey were not replaced during the experiment and we did not collect data on the predation sequence.

All individual predators and prey were used only once. Natural mortality of prey, evaluated in four control trials run in the experimental vessels without a predator using the same prey combination and density as in the experiments with predators, was low (Table 2). To account for its potential impact on the results, mean number of dead prey in control trials was subtracted from prey missing at the end of each experiment. No dead uneaten prey was found in the predation experiments suggesting that wasteful killing [33] did not occur.

All predators and 20 randomly chosen individuals of each prey species were preserved in 80% ethanol and their body length excluding appendages was measured to nearest 0.1 mm (Table 1 and 2). We also classified their microhabitat use during the experiments. Almost no individuals of any species used the perching sites except *Coenagrion* larvae. Only two microhabitats were thus recognized: water column (including perching sites) and bottom. We refer to the second- and third-instar beetle larvae as L2 and L3, respectively. Last-instar larvae of dragonflies and damselflies are referred to as F-0 and larvae of the second instar before the last as F-2. Instar numbers are omitted throughout the text if only one instar was investigated and names are abbreviated to the genus except if multiple species from the same genus are discussed.

### Data Analyses

Analyses were carried out and figures made in R 2.11.0 [34] unless stated otherwise. Selectivity of individual predators was evaluated using Manly’s selectivity index α [35], [36]:
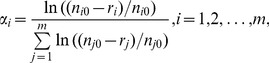
(1)where *n_i0_* is the initial number of prey items of type *i*, *r_i_* is the number of prey items of type *i* consumed by the predator and *m* is the number of prey types used in the experiment. Occasionally the predator consumed all individuals of the most preferred prey. To calculate Manly’s α in these cases, eq. (1) was modified by adding one individual of this prey to the corresponding *n_i0_* and *n_j0_*. This assumes that the added individual would have survived, and the corresponding estimate of α*_i_* is slightly conservative. Values of α*_i_* for individual prey species were compared with values expected for no selectivity using separate t-tests as recommended by Manly [37]. For presentation, values of α*_i_* were converted into electivity indices [36]. The indices for individual prey types range from −1 (prey absent in diet) to +1 (prey representing 100% of diet), with a value of 0 corresponding to unselective feeding. Diet breadth of a predator was defined as the number of prey types with electivity index larger than −1 (i.e. it only excluded prey that was never consumed). Numbers of prey consumed by individual predators and corresponding values of Manly’s alpha are shown in Table S1 (Supporting Information).

Pairwise diet overlaps of predators were calculated using Pianka’s overlap index [6] in Ecosim 7.0 [38]:
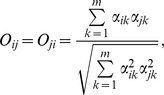
(2)where *O_ij_ = O_ji_* is the diet overlap between predator species *i* and *j* and symbol α*_pk_* denotes value of Manly’s *α* for prey type *k* consumed by predator *p* (*p = i* or *j*). Value of Pianka’s overlap index *O_ij_ = O_ji_* = 1 means that the diet of the two predators is identical; the lower the value, the less similar their diets. We also modified eq. (2) to calculate overlaps *P_ij_ = P_ji_* in predation pressure between prey species *i* and *j* by replacing Manly’s α with prey mortality:
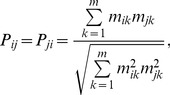
(3)where the mortality *m_ph_* of prey type *p* (*p* = *i* or *j*) consumed by predator *k* is calculated as the ratio of consumed prey individuals, *r_pk_*, over the initial number of prey, *n_p0_*. We used diet dissimilarity *D_ij_* = 1– *O_ij_* and a predator dissimilarity index *D_ij_* = 1– *P_ij_* as input data in Ward’s hierarchical clustering (stats package for R; [34]) and in nonmetric multidimensional scaling (NMDS, vegan 1.17-2 package for R; [39]) to classify and visualize the overlaps.

Finally, we analyzed the modularity of our experimental food web with two trophic levels to complement the analysis of similarities of predators based on their diet overlaps and similarities of prey based on shared predators. The aim of the analysis was to test whether our experimental food web consists of distinct modules characterized by numerous (strong) interactions within modules and few (weak) interactions among modules [22], [23], [40]. Our experiments provided us with quantitative data on the strength of predator-prey interactions, representing a weighted bipartite network. Hence, we used an algorithm for weighted networks [41] implemented in bipartite 1.17 package for R [42] to detect modules. Our experiments yielded two alternative measures of interaction strength, predator selectivity (Manly’s α) and prey mortality. We used both measures to assess the robustness of the results; the original data on the scale between 0 and 1 were multiplied by 100 and rounded to the nearest integer before each analysis.

### Review of Published Experiments

To complement our results, we reviewed previous laboratory experiments on prey selectivity with the same three groups of predators and various aquatic invertebrates used as live prey. We first searched Web of Science and Zoological Record using search phrases ‘predation AND taxon’, ‘foraging AND taxon’, ‘predator AND taxon’ and ‘prey AND taxon’, where ‘taxon’ stands for appropriate names of the predators and prey at various taxonomical levels. The results were complemented by a thorough search of the references in the relevant papers and of publications citing these papers. Only studies using more than one prey species for a predator were included, i.e. we omitted studies of stage or size selectivity. We also excluded studies dealing only with vertebrate prey (tadpoles and fish fry) and studies on cannibalism and intraguild predation. For each experiment, we noted the predator and prey taxa and developmental stages, experimental setup, method of data analysis and the main results (preferred prey or lack of selective feeding). We further classified the predators and prey as occupants of water column or the benthic microhabitat as in our experiment; we mostly used known information on their microhabitat use because most experiments did not specify this behaviour. We also noted the use of any habitat structure such as bottom substrate and natural or artificial vegetation to assess the impact of refuges and perching sites on the results.

To test which prey (at the level of order) are more preferred, we pooled all published experiments except those with prey from a single order and ranked each prey from the most to least preferred. The matrix of the prey ranks in each individual experiments based on the entire dataset had 85% empty cells as most experiments used only 2–3 prey types, thereby precluding the use of the method of analysis of incomplete ranking data as described in [43]. A necessary condition for a meaningful analysis is at least ∼50% non-empty cells [43], which we could achieve only by restricting the dataset to studies involving only Cladocera, Diptera and/or Ephemeroptera. However, these studies clearly showed that Diptera were more preferred than Ephemeroptera and slightly less preferred than Cladocera, making the analysis redundant. We therefore simply scored the preference for each taxon in each experiment on binary scale (1 = most preferred prey and 0 = all other prey in a given experiment) and compared the probability of being the most preferred prey taxon, using a generalized linear model with quasi-binomial distribution. This allowed us to compare the preferences across all prey taxa. We further used multiple comparisons of means for generalized linear models in multcomp package for R [44] to perform post-hoc pairwise comparisons of preference between different prey taxa.

To test for microhabitat association between predators and their preferred prey, we counted experiments that identified one or multiple prey from a single microhabitat (benthic/water column) as the most preferred and had at least one non-preferred prey from the other habitat. We used the resulting 2×2 contingency table to test the microhabitat association with a one-tailed Fisher’s exact test.

## Results

### Laboratory Experiment

We focus on the following six aspects of our experimentally assembled food web: selectivity of predators, diet overlaps of different predators, ontogenetic diet shifts, prey vulnerability, predator overlaps of different prey and food web modularity.

**Figure 1:**
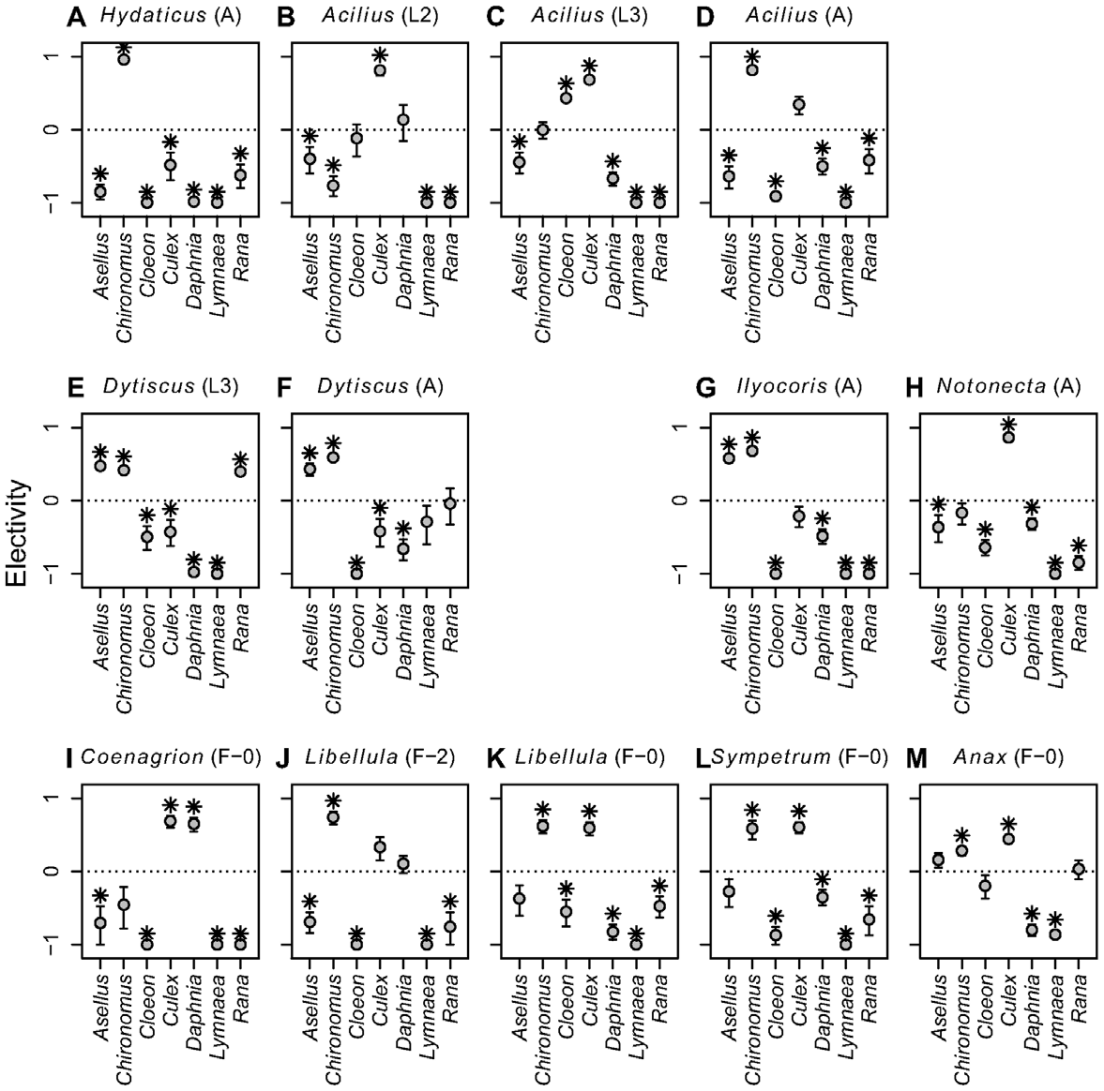
Prey selectivity of predatory aquatic insects. Mean values ± SE of electivity index are plotted. Positive values indicate preferred prey. Prey species with electivity values significantly different from zero (*P*<0.05, Holm’s correction of P-levels within each predator species was used) are marked by asterisk. Predator stage as in Table 1. Panels are sorted taxonomically: A–F = Coleoptera, G–H = Heteroptera and I–M = Odonata.

All predator species fed selectively but differed in their level of specialization (Figure 1). Adult diving beetles (*Acilius* and *Hydaticus*) and adult *Notonecta* were most selective, having only one preferred prey and at most one neutrally selected prey type (i.e., consumed proportionally to its abundance). Two more predators, *Libellula* F-2 and *Acilius* L2 larvae, were fairly specialized with one preferred prey and two prey with neutral preference. Most other predators (*Acilius* L3 larvae, *Dytiscus* and *Ilyocoris* adults and *Coenagrion*, *Sympetrum* and *Libellula* F-0 larvae) significantly preferred two prey and had neutral preference to one more prey type. *Dytiscus* and *Anax* larvae were least selective. *Dytiscus* larvae strongly preferred and nearly depleted three prey types (*Asellus*, *Chironomus* and *Rana*), while *Anax* larvae consumed five out of seven prey species at least proportionally to their abundance and significantly preferred two of them (*Chironomus* and *Culex*). Diet breadth was related to but not identical with the preference patterns. It ranged from all seven prey in *Anax* larvae to four prey in adult *Ilyocoris* and in *Coenagrion* larvae. Diet breadth of the other predators was five or six prey types.

**Figure 2:**
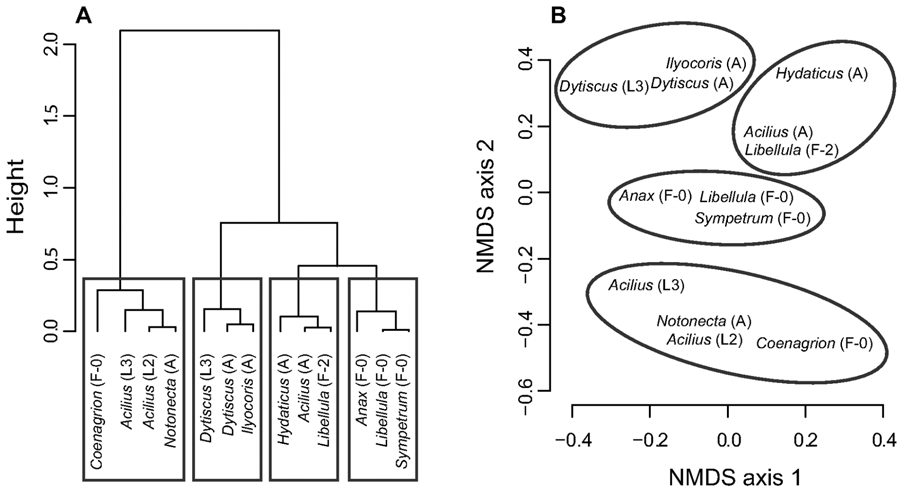
Similarity of diets of the predators used in the experiment. A. Ward’s hierarchical clustering of the diet dissimilarities *D_ij_*; height = value of clustering criterion for the particular cluster. B. Diet dissimilarities *D_ij_* visualised by nonmetric multidimensional scaling (NMDS, stress = 3.99), with groups identified by the cluster analysis highlighted; position of each species in the diagram corresponds to the centre of its label.

Consequently, diet overlaps of predator pairs varied greatly between 0.09 (*Acilius* L2 and adult *Hydaticus*) and 0.99 (*Sympetrum* and *Libellula* F-0; Table 3). Cluster analysis suggested four predator groups (Figure 2) with strong pairwise overlaps (≥0.80 except between *Coenagrion* and *Acilius* L3) within each group (Table 3). The first group comprises medium-sized benthic predators with strong preference for *Chironomus* and avoidance of *Asellus* (adult *Acilius*, adult *Hydaticus* and *Libellula* F-2 larvae; diet overlap 0.89–0.98). The second group consists of larger benthic odonate larvae which fed mainly on both species of dipteran larvae and neutrally selected *Asellus* (*Anax*, *Sympetrum* and *Libellula* F-0 larvae; diet overlap 0.89–0.99). Another group of large-bodied predators foraging on the bottom contains larvae and adults of *Dytiscus* and adult *Ilyocoris* (diet overlap 0.82–0.95). They all consumed large numbers of *Asellus* and *Chironomus*, although *Ilyocoris* did not feed on tadpoles. The fourth group (*Coenagrion* larvae, L2 and L3 larvae of *Acilius* and adult *Notonecta*; diet overlap 0.66–0.97) foraged mainly in the water column and near the surface and fed mainly on *Culex* or, as in *Coenagrion* larvae, on *Daphnia* and *Culex*. We call these predators nektonic hereafter.

**Table 3:** Pairwise diet overlaps of predators.

	Hydaticus(A)	Acilius(L2)	Acilius(L3)	Acilius(A)	Dytiscus(L3)	Dytiscus(A)	Ilyocoris(A)	Notonecta(A)	Coenagrion(F-0)	Libellula(F-2)		Libellula(F-0)	Sympetrum(F-0)
Acilius (L2)	0.09												
Acilius (L3)	0.30	**0.89**											
Acilius (A)	**0.94**	0.41	0.55										
Dytiscus (L3)	0.59	0.21	0.35	0.64									
Dytiscus (A)	0.78	0.21	0.35	**0.80**	**0.92**								
Ilyocoris (A)	0.78	0.26	0.39	0.79	**0.82**	**0.95**							
Notonecta (A)	0.21	**0.97**	**0.87**	0.52	0.28	0.30	0.34						
Coenagrion (F-0)	0.14	**0.88**	0.66	0.41	0.17	0.22	0.27	**0.80**					
Libellula (F-2)	**0.89**	0.50	0.57	**0.98**	0.58	0.76	0.78	0.57	0.57				
Libellula (F-0)	0.76	0.69	0.78	**0.93**	0.61	0.72	0.73	0.79	0.58		**0.91**		
Sympetrum (F-0)	0.72	0.74	0.77	**0.91**	0.59	0.71	0.73	**0.82**	0.67		**0.92**	**0.99**	
Anax (F-0)	0.56	0.72	**0.81**	0.76	**0.80**	0.78	0.74	0.78	0.57		0.74	**0.90**	**0.89**

Overlaps calculated as Pianka’s index (eq. 2); values larger than or equal to 0.80 shown in bold. Predator stages as in Table 1.

We tested for an ontogenetic diet shift (ODS) in two diving beetles (L2 and L3 larvae and adults of *Acilius*, L3 larvae and adults *Dytiscus*) and one dragonfly (F-2 and F-0 larvae of *Libellula*). In all three species, diets differed significantly between the stages (Table 4 and Figure 1). Significant diet shifts occurred mainly in the preferred prey. We observed strong ODS in *Acilius* from *Culex* (preferred by L2 larvae) to *Cloeon* and *Culex* (preferred by L3 larvae) and subsequently to *Chironomus* (preferred by adults). Moreover, adult *Acilius* also fed on *Rana* tadpoles, which were never eaten by the larvae. Diet overlap was therefore much lower between the adults and larvae (0.41 and 0.55) than between the two larval instars (0.89; Table 3). Diet overlaps within the other two species were high (0.91 and 0.92) and the resulting ODS mainly quantitative (Figure 1).

**Table 4:** Tests of ontogenetic diet shifts (pairwise comparisons based on t-test).

	*Acilius*adult vs. L3		*Acilius*adult vs. L2		*Acilius*L3 vs. L2		*Dytiscus*adult vs. L3		*Libellula*F-0 vs. F-2	
Prey	t	*P*	t	*P*	t	*P*	t	*P*	t	*P*
*Asellus*	−0.93	0.37	−0.99	0.34	−0.20	0.84	−0.46	0.65	1.17	0.28
*Chironomus*	7.72	**2•10^−5^**	10.42	**<10^−5^**	4.00	0.002	2.58	**0.03**	−0.96	0.36
*Cloeon*	−11.15	**<10^−5^**	−2.42	0.051	3.50	**0.006**	−2.41	0.07	2.01	0.10
*Culex*	−3.28	**0.006**	−3.43	**0.007**	−1.47	0.18	0.03	0.98	1.58	0.14
*Daphnia*	1.18	0.26	−1.75	0.13	−2.05	0.08	1.90	0.09	−4.40	**0.003**
*Lymnaea*	–	–	–	–	–	–	1.93	0.09	–	–
*Rana*	2.66	**0.03**	2.66	**0.03**	–	–	−2.61	**0.03**	1.14	0.28

Positive and negative t-values respectively mean that the prey is more preferred by later and earlier predator stage (e.g., adult *Acilius* prefer *Chironomus* more and *Culex* less than *Acilius* L3 larvae do); see also Fig. 1. Missing results (−) indicate that neither stage consumed the prey. Significant results (*P*<0.05) are highlighted in bold.

**Figure 3:**
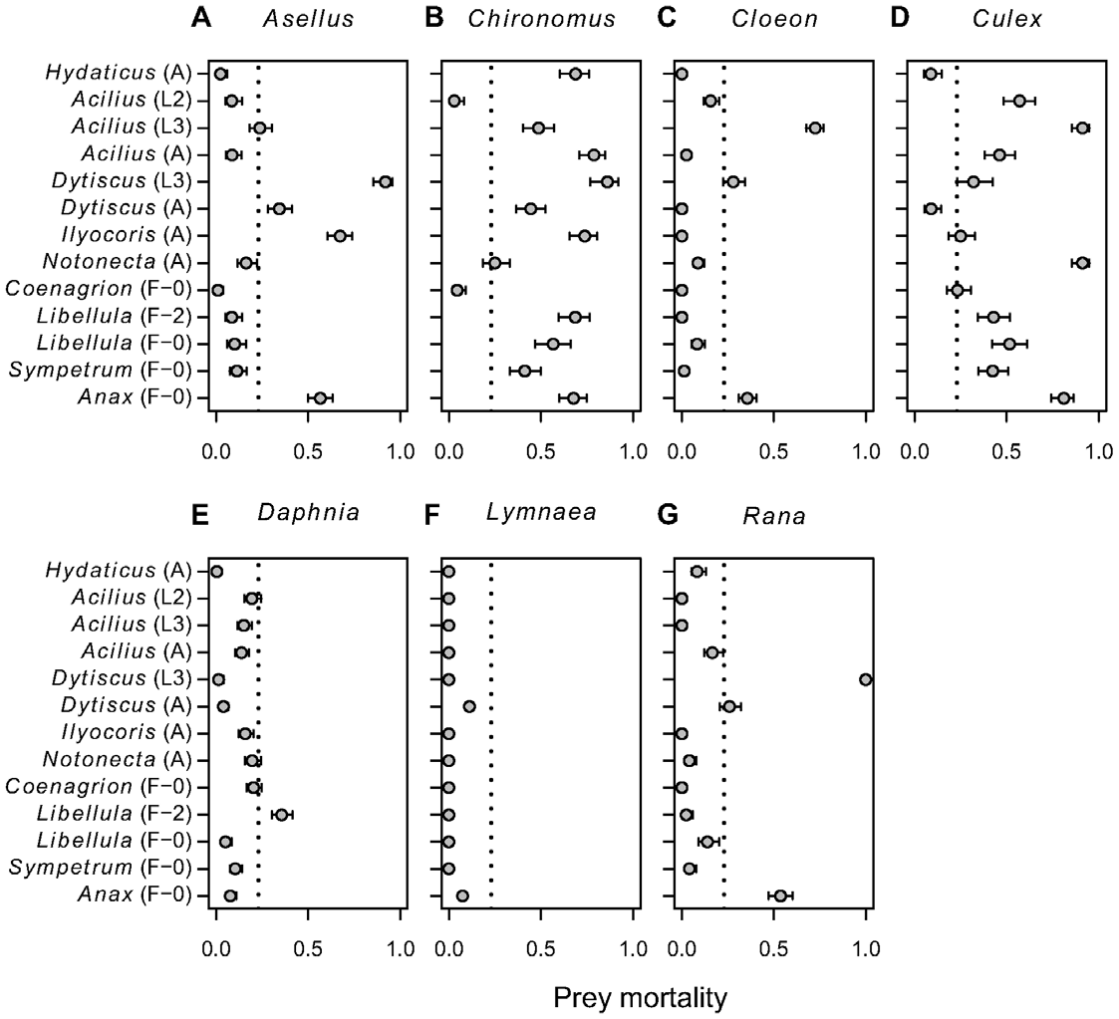
Mortality of individual prey species subjected to different predators. Prey mortality is expressed as proportion of individuals eaten during the experiment (mean ± SE). Dotted vertical lines represent the overall observed mortality averaged across all combinations of prey and predator species. Predator stage as in Table 1; predators ordered taxonomically as in Table 1 and Figure 1.

Vulnerability of each prey species to predation differed significantly across all predators (proportion of prey individuals consumed during experiment; GLM with quasi-binomial distribution, *P*<0.0001 in all cases; Figure 3). *Chironomus* and *Culex* were most vulnerable overall (average mortality 51% and 46%, respectively). Either of them was the most preferred prey for each predator (Figure 1) except *Dytiscus* larvae. Three other species were highly vulnerable only to a subset of predators (mortalities of *Rana* tadpoles: 100% from *Dytiscus* larvae, 54% from *Anax* larvae; *Asellus*: 92% from *Dytiscus* larvae, 68% from *Ilyocoris*; *Cloeon*: 73% from *Acilius* L3 larvae). The least consumed prey was *Lymnaea*, eaten only by *Anax* larvae and adult *Dytiscus* (ca. 10% mortality from either predator; Figure 3).

**Figure 4:**
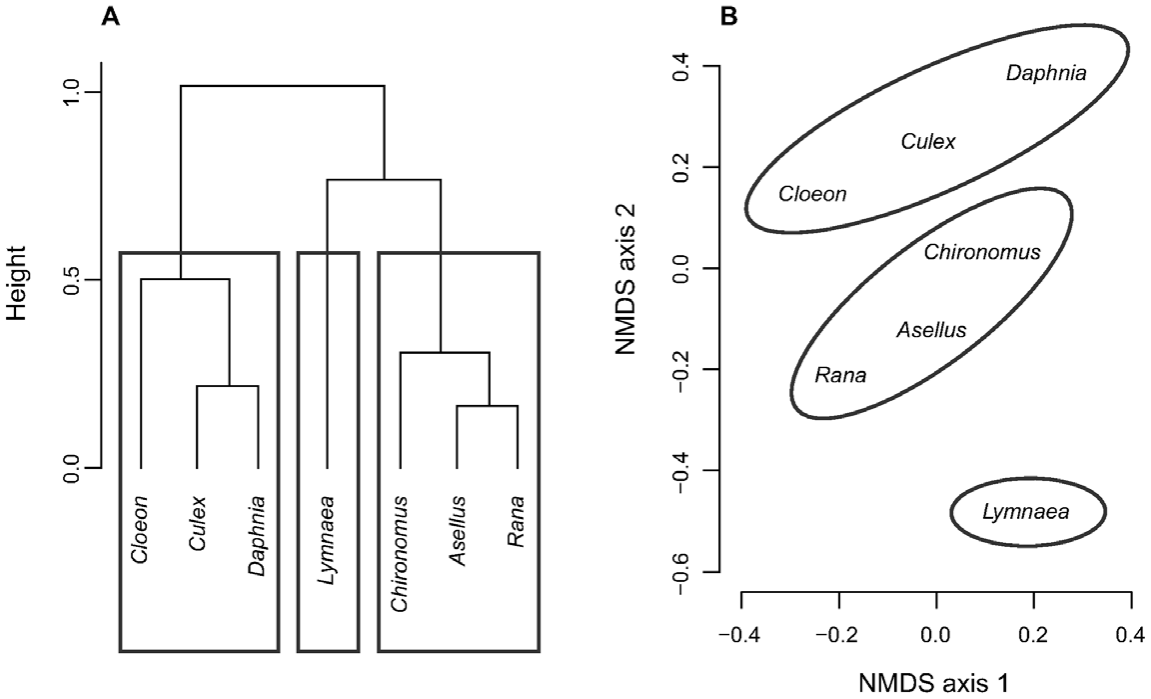
Similarity of predation pressure among prey species used in the experiment. A. Ward’s hierarchical clustering of the dissimilarities of predation pressure *D_ij_*; height = value of clustering criterion for the particular cluster. B. Predation pressure dissimilarities *D_ij_* visualised by nonmetric multidimensional scaling (NMDS, stress = 2.29), with groups identified by the cluster analysis highlighted; position of each species in the diagram corresponds to the centre of its label.

Predator overlaps among prey species varied between 0.13 (*Daphnia* and *Lymnaea*) and 0.83 (*Asellus* and *Rana*; Table 5). Cluster analysis identified three prey groups (Figure 4): the largely invulnerable *Lymnaea*, one group of larger benthic prey with mostly shared predators (*Rana*, *Asellus* and *Chironomus*; predator overlap 0.67–0.83) and another group of smaller, non-benthic prey (*Cloeon*, *Culex* and *Daphnia*; predator overlap 0.39–0.78). Pairwise overlaps in predators within the prey groups were thus on average lower than diet overlaps within the predator groups.

**Figure 5:**
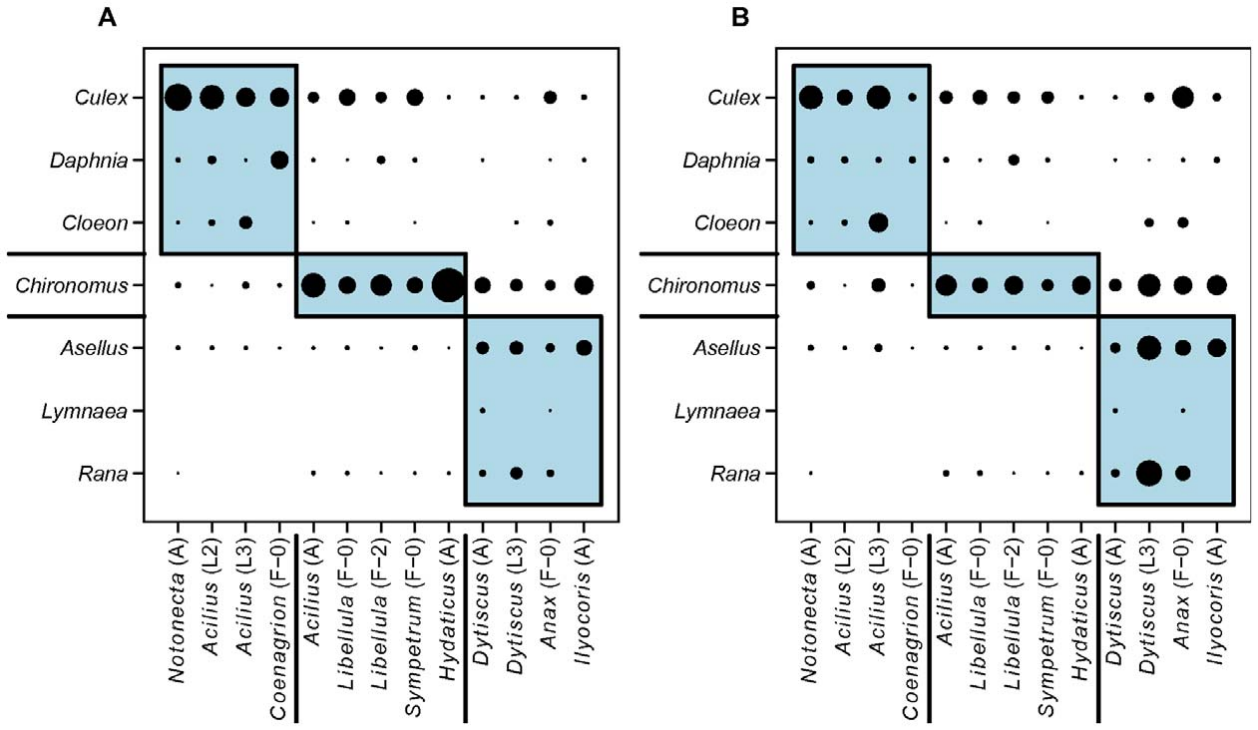
Modularity of the experimental food web. Modules identified by modularity analysis displayed as boxes; symbol size corresponds to predator-prey interaction strength. A. Predator preference (Manly’s α) used as measure of interaction strength. B. Prey mortality used as measure of interaction strength.

Finally, modularity analysis identified three modules in our experimental food web. A nektonic module containing four predators (*Notonecta*, L2 and L3, *Acilius* larvae and *Coenagrion*) and three prey (*Daphnia*, *Cloeon* and *Culex*) is identical to the combination of the respective predator and prey groups identified by cluster analysis (Figures 2 and 4). The other two modules involve benthic prey and predators (Figure 5). Both measures of interaction strength yielded the same results (Figure 5A and 5B), suggesting that the conclusions are robust. In addition, predators in the two “benthic” modules correspond well to the results of the cluster analysis, which subdivided one of the modules into two clusters and otherwise assigned only one species (*Anax*) differently (Figures 2 and 5). The benthic prey modules differ from the results of cluster analysis only by isolating the strongly linked *Chironomus* rather than excluding the weakly linked *Lymnaea* from the remaining three benthic prey (Figures 4 and 5).

### Review of Published Experiments

Thirty-five studies reporting 59 experiments with more than 40 predator species satisfy the predefined criteria (Supporting Information Table S2). Similar numbers of experiments used diving beetles (*n* = 19), bugs (*n* = 22) and odonate larvae (*n* = 18) but one model taxon prevails in each group: *Dytiscus* in diving beetles, *Notonecta* in bugs and *Anax* in dragonflies. The prey included more than 50 species ranging from protozoans to amphibians. Surprisingly rare were studies in which the prey included Isopoda (four experiments), Oligochaeta (one) and studies comparing invertebrate and vertebrate prey (five). Prey composition varied greatly among studies and rarely was diverse enough to represent a semi-natural mixture. Up to 21 (mostly 2–6) prey types were offered; more than six prey types usually involved experiments with some prey types representing multiple size classes of the same species.

**Table 5:** Pairwise overlaps of predator assemblages associated with different prey species.

	Asellus	Cloeon	Culex	Daphnia	Chironomus	Lymnaea
Cloeon	0.56					
Culex	0.59	**0.75**				
Daphnia	0.41	0.39	**0.78**			
Chironomus	**0.79**	0.51	**0.73**	0.64		
Lymnaea	0.44	0.22	0.27	0.13	0.36	
Rana	**0.83**	0.47	0.44	0.17	0.67	0.43

Overlaps calculated as Pianka’s index (eq. 2); values larger than 0.70 shown in bold.

**Figure 6:**
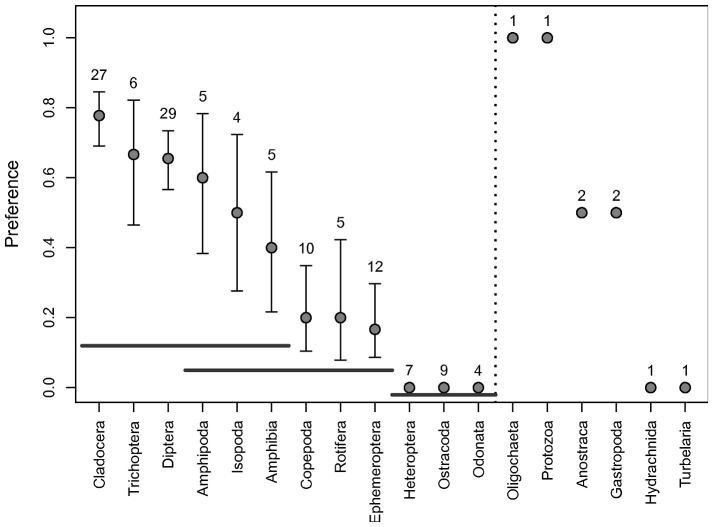
Relative vulnerability of prey taxa most often used in previous experiments. Based on data in Supporting Information Table S1. Preference = proportion of experiments in which the prey taxon was most preferred (mean ± SE). Number of experiments that included a given prey taxon is stated above each error bar. Dotted line separates prey types used in >3 experiments (to the left) and <3 experiments (to the right). Horizontal grey lines denote prey groups that do not differ significantly according to multiple comparisons of means (*P*>0.05).

Almost all papers reported distinct selectivity of the predator towards some of the prey (Supporting Information Table S2). Taken together, they reveal large and significant differences between preferences for different prey taxa (GLM with quasi-binomial distribution, F = 5.01, *P* = 0.00003; Figure 6). Cladocera were most preferred in 21 out of 27 experiments in which they were used together with alternative prey from a different order; most of these experiments used *Daphnia* (preferred in 19 out of 22 experiments). Various dipterans were also frequently preferred (19 out of 29 experiments); larvae of Culicidae were favoured in 12 out of 28 experiments and Chironomidae in 10 out of 13 experiments (in six of these cases, both families were tested together). Ten other prey taxa were preferred in at least one experiment, with Copepoda (two of 10 cases), Rotifera (one of four cases) and Ephemeroptera (two of 12 cases) among the least preferred. Five taxa were never preferred: Heteroptera (*n* = 7 experiments), Ostracoda (*n* = 6), Odonata (*n* = 4), Hydrachnida (*n* = 1) and Turbellaria (*n* = 1). Post-hoc pairwise comparisons of the simplified data on predator preferences suggested that (i) the preference for the three taxa never preferred in multiple experiments is significantly lower (t-test, *P*<0.05) than for the remaining taxa and (ii) the preference for Cladocera, Trichoptera and Diptera is significantly higher than that for Copepoda, Rotifera and Ephemeroptera (Figure 6). Due to the small sample sizes, we could not compare if the preferences differed among predator taxa or if a pre-existing bias in the selection of prey combinations affected the results.

Among 33 experiments with benthic predators, benthic prey was preferred over prey in the water column in 10 experiments and the reverse was found in 4 experiments; the remaining experiments used only prey occupying a single microhabitat or the experiment found conditional preference for either type of prey. The proportion of clear outcomes was even lower among the 24 experiments with predators foraging in the water column, with one and three experiments respectively reporting preference for benthic and nektonic prey. This is suggestive of an overall preference for prey from the same microhabitat, but the results are inconclusive (*P* = 0.14, one-tailed Fisher’s exact test).

Taken together, the results of previous experiments show that larvae of dragonflies and damselflies, aquatic bugs and diving beetles are selective predators. In general, they seem to feed most heavily on cladocerans and on the larvae of Diptera (mosquitoes and chironomids). The preferences are at least partly driven by overlapping microhabitat use: predators preferring cladocerans and mosquito larvae (e.g., *Notonecta*) usually forage in the water column, while chironomid larvae are generally preferred by benthic predators (Supporting Information Table S2).

## Discussion

### Diet overlaps and Coexistence of Multiple Predators

Predators that coexist in the same habitat need to occupy different space, time and/or food niche [6], [45]. Resource partitioning and restricted diet overlap leading to distinct food niches supposedly drive predator coexistence in lizards (e.g., [6], [45]), fish [46], [47] and carnivorous mammals [48], [49], although some recent studies question the importance of food niche partitioning (e.g., [50]). Data on invertebrates are scarcer and more controversial (e.g., [51]–[54]) and lacking for aquatic insects in standing waters. Although the link between niche separation and coexistence should be rigorously tested [55], measuring diet overlaps among co-occurring species provides important insights into the potential contribution of resource partitioning to long-term coexistence.

Our experimental food web consisted of three modules determined by individual body size and microhabitat use. Nektonic predators that utilized mainly the water column (*Acilius* and *Coenagrion* larvae and *Notonecta*) and fed mainly on nektonic prey (*Daphnia* and *Culex*) had limited diet overlap (0.09–0.82, mean 0.47) with bottom-foraging “benthic” predators (adult diving beetles, *Ilyocoris* and *Libellula* and *Dytiscus* larvae; Figure 2). On the contrary, predators sharing the same microhabitat had a strong, typically >0.80 overlap in diets. This result likely holds across taxa. For example, diet preferences of other nektonic backswimmers (genera *Anisops* and *Buenoa*) are similar to *Notonecta*, while the more sedentary bugs from the families Pleidae, Belostomatidae and Naucoridae seem to prefer benthic prey (Supporting Information Table S2).

A formal test supporting the idea of microhabitat-use driven modularity by the literature data was inconclusive, mainly because many previous experiments, especially with nektonic predators, used prey from only one microhabitat. Among the four nektonic predators with a clear preference, only larvae of *Acilius* preferred benthic prey [56]. This could have arisen if the individual predators perceived the benthic habitat and water column as one habitat, e.g. in shallow water, and chose prey according to some other criteria. We could not assess this phenomenon due to paucity of data; the impact of water depth on diet overlaps between nektonic and benthic predators deserves further study.

Furthermore, large predators are probably less constrained by microhabitats than small-bodied predators, and their diet is driven primarily by high metabolic demands and the need for high energy intake rates. Feeding links of such predators may thus provide connections between separate food web modules. In our experiments, *Anax* larvae were the least selective and consumed all prey species as the only predator. Overall, larvae of diving beetles (*Dytiscus*) and dragonflies (*Anax*) are known as voracious predators of tadpoles [30]–[32], [57]–[59] and other large prey including smaller conspecifics and intraguild prey [60]–[63]. This effect may not be universal: some *Dytiscus* species have a specialized diet, such as large caddisfly larvae [30], [58].

Prey selectivity of smaller predators has been less studied (Supporting Information Table S2). It is incompletely understood apart from damselflies, which are known to feed mostly on zooplankton and are thus linked within a nektonic module ([20], [64]; references in Supporting Information Table S2). Only few studies focused on the diets and prey selectivity of medium-sized dragonflies, which are among classic taxa used in various ecological experiments (Supporting Information Table S2). The two species in our study, *Libellula* and *Sympetrum*, fed mainly on smaller benthic prey (*Chironomus*) and mosquito larvae, which is in line with previous results [56].

In other words, similarly sized predators foraging in the same microhabitat will often–but not always–have strongly overlapping diets. These predators are “generalists in a narrow sense”, i.e. have a broad diet conditional on their size and foraging microhabitat, and can be subject to intense indirect competitive interactions with potentially fundamental consequences for the entire food web structure. For example, food depletion in predatory aquatic insects may increase cannibalism and intraguild predation [60], which are both common among larvae of diving beetles [63] and odonates [60]–[62]. Intraguild predation of dytiscid larvae by odonates may cause negative correlations between odonate and dytiscid densities [65]. However, indirect competitive interactions are difficult to measure in the field and have been reported by very few studies on predatory aquatic insects. Data in [66] suggest that adult diving beetles are not food limited and hence protected from exploitative competition. That study found significant density-dependent mortality, possibly caused by competition for food or cannibalism, only in the larvae.

Diet overlaps have not been formally calculated in predatory aquatic insects before. Values found in our experiment mostly fall within the range known in other taxa, although Pianka’s indices of 0.66–0.99 (mostly≥0.87) within each of the predator clusters identified in our experiment are unusually high. Such nearly complete diet overlaps are uncommon in both terrestrial and aquatic vertebrate predators (e.g., [48], [67]–[70]), and overlap index as low as 0.76 has been implicated in species replacement driven by food competition [71]. Coexistence of predators with so strongly overlapping diets requires additional mechanisms such as exploitation of different size classes or stages of the shared prey [72], [73].

The observed overlaps would likely decrease with a broader range of prey species that are unfeasible to test in the laboratory, but we believe that the decrease would be limited given the broad coverage of prey sizes and functional types. Moreover, we have tested only one size class for each prey and thus cannot establish if size selectivity or other factors–such as differences in the diet concerning prey not included in the experiments, different time and/or space niches, and strong intraspecific competition or cannibalism–help these predators coexist in the same habitat. The importance of apparent competition and interference mediated by overlapping diets for population dynamics of predatory aquatic insects thus requires further study.

Coexistence on local spatial scales, e.g. through diversification of diets, might also lead to coexistence at larger scales. We speculate that this mechanism could contribute to high regional diversity of diving beetles relative to the other predators (e.g., Czech Republic: ∼120 species of diving beetles, ∼60 species of odonates and ∼40 species of aquatic bugs; [74], [75]). For example, we found that adult *Hydaticus* consumed mainly *Chironomus* larvae, while experiments with other similarly sized genera and different prey sets found preference for *Daphnia* [19]. Larvae of *Agabus* and *Acilius* are efficient predators of mosquito larvae [76], [77], which is consistent with our results on *Acilius canaliculatus*. Some diving beetles even prefer dead prey [78]. They may be opportunistic scavengers that exploit yet another food niche.

### Ontogenetic Diet Shifts

The concept of ontogenetic diet shift (ODS) is rapidly becoming a central theme in studies of aquatic food webs. Most animals grow substantially during their development and body size is now recognized as a key driver of predator-prey relationships, particularly in the aquatic environment [2], [79]. Predation pressure on prey assemblages can thus change considerably as the predators grow, which can have both ecological and evolutionary consequences [80]. In addition, diet shifts may release individual predators from intraspecific competition for food in the same way as reduced diet overlaps decrease apparent competition between species. ODSs are well documented in various holometabolous taxa with complex life histories [81]. Shifting diets may also reflect changes in foraging (micro)habitat and behaviour. They occur in *Notonecta* bugs [82]–[84] and in larval odonates in both running [24] and standing waters [18]. Odonate larvae begin to feed on rotifers and even protozoans after hatching [85]–[87] and later switch to larger benthic prey.

We detected more or less pronounced ODSs in all three predators for which we tested more than one life stage (*Acilius*, *Dytiscus* and *Libellula*), indicating that ODSs are widespread in predatory aquatic insects. ODSs should be particularly common in larvae and adults of diving beetles, which differ greatly in morphology, foraging habitat and behaviour. Surprisingly, observed diet overlaps between larvae and adults (∼0.4–0.5 in *Acilius* and ∼0.9 in *Dytiscus*) were quite high. Diet overlaps between last-instar larvae and adults calculated as in [81] (0.42 in *Acilius* and 0.29 in *Dytiscus*) greatly exceed the previously reported range (0–0.08) for metamorphic species and are closer to the values typically ascribed to continually growing, gape-limited predators [81]. This suggests that limited diet overlaps may require non-overlapping habitats (as in odonates) or the presence of other mechanisms absent in the diving beetles. Diet shifts occurred also between consecutive larval instars (*Acilius* and *Libellula*), even if they were smaller and the overlaps in diet (∼0.9) fell within those observed in the four predator clusters.

We conclude that ODSs in predatory insects in small fishless pools sometimes allow species to move between food web modules during ontogeny (larvae and adults of *Acilius*). However, ODSs may not always be strong enough to release successive instars/stages from intraspecific competition and the predator may remain in one food web module throughout most of its aquatic life (as in *Dytiscus* and larvae of *Libellula*). Further experiments and observations are needed to quantify the overlaps across multiple instars/stages for multiple predators and resolve this issue.

### Vulnerability of Prey Species to Predation

Last but not least, we showed that vulnerability to predation varies greatly among common prey found in fishless pools and other standing water bodies of the temperate zone in Europe. The most vulnerable prey were dipteran larvae (*Culex* and *Chironomus*), which were also preferred by predators in most other experiments. In addition, the literature review suggested that cladocerans, especially *Daphnia*, are the most preferred prey of predatory aquatic insects along with trichopteran and dipteran larvae. In our experiment, cladocerans were rarely preferred. This discrepancy most likely reflects different prey composition in the experiments. Previous studies often coupled *Daphnia* with other zooplankton and thus probably offered a suboptimal prey choice to many predators. Neither have we tested some of the prey reported as non-preferred in previous experiments (Copepoda and Ostracoda; probably invulnerable or non-profitable to most predatory aquatic insects). In addition, some predator-prey combinations were rare in previous experiments. Suggested overall ranking and differences in preferences between the prey (Figure 6) must be therefore taken cautiously as many experiments probably pre-selected prey taxa to test *a priori* hypotheses.

Prey vulnerability is partly determined by its ability to withstand or avoid predator attacks. *Culex* larvae are capable of rapid escape movements in the water column, but these were apparently not effective against most predators in our experiment as the predation rates were high. High vulnerability of *Culex* larvae to various invertebrate predators was repeatedly confirmed in experiments aimed to identify potential anti-malaria control agents [88]. Given their frequently high population densities (e.g., [76]), mosquito larvae possibly serve as keystone prey species that support a large number of different predator species and contribute to the maintenance of high species diversity in small fishless standing water bodies.

*Chironomus* larvae were exposed and lacked refuge in our experiment, and hence represented highly profitable and easily accessible prey (see Supporting Information File S1 for full discussion of the methodological issues associated with most multiple-choice predation experiments). Cothran & Thorp [89] showed that the presence of a refuge can strongly decrease predation on chironomid larvae as the predator’s selectivity shifts towards alternative prey. Under natural conditions, chironomid larvae burrow in soft sediments to avoid predators and are vulnerable only when migrating [90]. At the same time, predators can successfully specialize on chironomids [90]. Our experimental setup thus corresponds to the presence of a large chironomid population with sufficiently many larvae available to predators. Alternatively, high preference for chironomid larvae in an experiment lacking refuges can indicate preference for benthic prey with high probability of successful attack and high profitability (such as any injured/diseased animals), or tendency towards facultative or obligatory scavenging, which could be the preferred feeding strategy for some predators [78].

On the other hand, prey vulnerability is greatly reduced by reaching a size refuge and/or mechanical defences (such as external hard shell), although some predators may adapt their foraging strategy to overcome the defences (e.g., *Dytiscus* preying on caddisflies; [58]). In our experiment, *Lymnaea* snails were the only protected and also the least vulnerable prey. In addition, we used relatively large snails that have apparently reached a size refuge, as small snails are vulnerable to predation [91]. Size refuge, along with species-specific diets or different size and identity of the prey assemblages, could also explain the observed lack of preference of adult *Dytiscus marginalis* for snails in our experiment, contrary to *Dytiscus alascanus* [21]. Similarly, *Rana* tadpoles lack mechanical defence but were the second least consumed prey overall, apparently as they were too large and difficult to handle for most predators except the largest ones (*Anax* and *Dytiscus*; compare [31], [57], [59]).

### Conclusions

Predatory aquatic insects of standing waters were often seen as generalists in the past [14]–[16]. This traditional view must be revised: the unfolding story on predator-prey interactions in standing fishless waters is one of complex, challenging patterns. Diets of predatory insects in these habitats vary from highly specialized to broadly general, but hardly any species appear to feed indiscriminately. By combining a simple experiment with a literature survey, we provide a basis for future studies on food webs involving predatory aquatic insects in small standing water bodies.

We found a highly interconnected experimental food web, separable into several modules based on microhabitat use (bottom or water column) and body size of the predators and their prey. We thus suggest that predatory aquatic insects in small standing water bodies are “generalists in a narrow sense”: species with similar size foraging in the same microhabitat have widely overlapping diets. Moreover, ontogenetic diet shifts associated with individual growth in size and changes in foraging microhabitats seem common across all major groups of predatory aquatic insects. That is, food web interactions of predatory aquatic insects might be equally affected by intraspecific and interspecific differences.

All these results have potentially crucial implications for the structuring and stability of food webs. We thus call for further predation experiments using artificially assembled food webs, in combination with other methods such as gut content analyses wherever applicable. Particularly needed are more systematic studies of ontogenetic niche shifts, studies of unlikely and random predator-prey combinations, which could resolve the otherwise overlooked issue of avoided prey or reveal unexpected predator-prey links, and comparative studies of prey preferences in the presence and absence of (semi-realistic) habitat structure. Only such pluralistic approach can map the structure of food webs in small standing waters.

## Supporting Information

Table S1 **Summary of the results of our experiments.** Initial number of prey, number of prey consumed and Manly’s alpha is shown for all experiments. The results of each experiment, identified by the number in “Predator ID” column, are presented in seven rows, one for each prey.
(XLS)

Table S2 **Summary of published experiments on selective predation by diving beetles, water bugs and larvae of odonates.** Only studies with more than one prey species for a predator are included, and studies which focused primarily on vertebrate prey and studies on cannibalism and intraguild predation are excluded. Each row corresponds to one experiment or a set of experiments with the same set of species, possibly with different size classes/developmental stages or densities. Stage: A = adults, L = larvae (number of instars or size classes given in parentheses). Prey No. = total number of prey types (species and size classes) used (superscripts: a = multiple experiments with prey in different combinations; b = multiple experiments with the same prey combinations but different prey abundances). Multiple choice = more prey species were offered simultaneously; yes/no means that some experiments were conducted with individual prey types separately (typically to study single prey functional responses) and other experiments with a mixture of prey. Test of selectivity: Roger = Roger’s index (Lundkvist et al., 2003) and mortality = comparison of prey mortalities or numbers of prey consumed by the predator. The most preferred prey is highlighted in bold. When the most preferred prey differ between treatments (in experiments which offered prey in different combinations/abundances), all of them are highlighted. Bottom structure presence: yes/no = manipulated presence/absence. Preferred microhabitat of predators and prey was classified into two categories: water column and benthic. References appearing only in Table S2 are listed in File S1.
(XLS)

File S1 **Multiple-choice experiments: theoretical background.** Summary of main conceptual issues related to our and previous experiments (including references) and references appearing only in Table S2.
(DOC)
