# Supplementary material for: Who Eats Whom in a Pool? A Comparative Study of Prey Selectivity by Predatory Aquatic Insects
Source: PLoS One. 2012 Jun 5;7(6):e37741. doi: 10.1371/journal.pone.0037741 (PMC3367957; doi:10.1371/journal.pone.0037741)
Supplement: File S1 — Multiple-choice experiments: theoretical background. Summary of main conceptual issues related to our and previous experiments (including references) and references appearing only in Table S2. (DOC) [file pone.0037741.s003.doc]

**Supplementary Information File S1**

**Who eats whom in a pool? A comparative study of prey selectivity by predatory aquatic insects**

**Jan Klecka ^1,2*^ and David S. Boukal ^1,2^**

^1^ Department of Ecosystems Biology, Faculty of Science, University of South Bohemia, Branišovská 31, České Budějovice, 37005, Czech Republic

^2^ Laboratory of Theoretical Ecology, Biology Centre of the Academy of Sciences of the Czech Republic, v.v.i, Institute of Entomology, Branišovská 31, České Budějovice, 37005, Czech Republic

* author for correspondence:

Jan Klecka, Department of Ecosystems Biology, Faculty of Science, University of South Bohemia, Branišovská 31, České Budějovice, 37005, Czech Republic, E-mail: [kleckj01@prf.jcu.cz](mailto:kleckj01@prf.jcu.cz)

**Contents:**

***1. Multiple-choice experiments: theoretical background.*** Summary of main conceptual issues related to our and previous experiments (including references).

***2. References appearing only in Table S2.***

**1. *Multiple-choice experiments: theoretical background***

Multiple-choice experiments are routinely used to study selective predation and have well known pros and cons [1]. An important advantage is that predators interact with prey in a more natural setting than in single-prey experiments. However, analysis and interpretation of the results relies on the assumption that predator selectivity is independent of prey depletion [2]. Prey depletion could have affected mainly the least selective predators in our experiment (larvae of *Dytiscus* and *Anax*), because they could have consumed otherwise ignored prey, which would lead to an overestimated diet breadth. On the contrary, optimally foraging predators could appear as obligate specialists if their preferred prey remains abundant [3]. Optimal foraging and other mechanisms may also lead to frequency-dependent prey selection [4,5], found in some predatory aquatic insects for particular combinations of prey types [6–8] but not for others [8–10]. However, “standard” optimal foraging theory often does not apply to consumers feeding on mobile prey, including most predatory aquatic insects [5].

The results may further become biased if functional responses vary between prey taxa. Given that structured habitats and refuges commonly generate type III functional responses (e.g., [11]), we used a simple experimental arena to minimize this effect at the expense of reduced realism of the experiment. Moreover, the prey differed in their preference for shelters: while benthic prey such as chironomid larvae would hide in the substrate, pelagic prey and prey associated with submerged plants require patches of macrophytes as refuges (see the section on prey vulnerability below for details). This problem is inherently multi-dimensional and cannot be solved by a single study.

Previous experiments varied in their treatment of habitat structure. Most of them (35 out of 59) used no habitat structure at all. The remaining experiments provided only simple structure on the bottom (layer of sand, stones or a few tree leaves) or in the water column (live submerged plants or artificial structures such as in our experiment), and hence were far from natural setting. Only five experiments used both bottom substrate and vegetation simultaneously, and only one study compared the presence and absence of habitat structure [12]. Structured habitat was most often used in experiments with diving beetles, while experiments with heteropterans and odonates were mostly conducted without structure.

In summary, our setup fulfils the standards followed by the vast majority of previous experiments. This setup focuses on multiple prey types and is open to bias stemming from the lack of multiple levels of prey densities or interactions between predators. We deliberately fixed the densities at values reasonably close to those observed under field conditions (Klecka & Boukal, unpublished data). This approach yields comparable results across predators and provides initial insights into the strength of predator-prey interactions in the field. Extending these experiments to measure multispecies functional responses (e.g., [13]), especially in contrasting environmental settings that would provide refuges for some/all prey types, would be highly laborious but could provide an avenue to scale up lab experiments to field conditions.

**References**

1. Manly BFJ (1995) Measuring selectivity from multiple choice feeding-preference experiments. Biometrics 51: 709–715.

2. Chesson J (1983) The estimation and analysis of preference and its relationship to foraging models. Ecology 64: 1297–1304.

3. Schoener TW (1971) Theory of feeding strategies. Annu Rev Ecol Syst 2: 369–404.

4. Sherratt T, Harvey I (1993) Frequency-dependent food selection by arthropods: a review. Biol J Linn Soc Lond 48: 167–186.

5. Sih A, Christensen B (2001) Optimal diet theory: when does it work, and when and why does it fail? Anim Behav 61: 379–390.

6. Akre BG, Johnson DM (1979) Switching and sigmoid functional response curves by damselfly naiads with alternative prey available. J Anim Ecol 48: 703–720.

7. Bergelson J (1985) A mechanistic interpretation of prey selection by *Anax junius* larvae (Odonata: Aeschnidae). Ecology 66: 1699–1705.

8. Saha N, Aditya G, Saha GK, Hampton SE (2010) Opportunistic foraging by heteropteran mosquito predators. Aquatic Ecol 44: 167–176.

9. Colton T (1987) Extending functional response models to include a second prey type: an experimental test. Ecology 68: 900–912.

10. Blois-Heulin C, Crowley PH, Arrington M, Johnson DM (1990) Direct and indirect effects of predators on the dominant invertebrates of two freshwater littoral communities. Oecologia 84: 295–306.

11. Vucic-Pestic O, Rall BC, Kalinkat G, Brose U (2010) Allometric functional response model: body masses constrain interaction strengths. J Anim Ecol 79: 249–256.

12. Cothran ML, Thorp JH (1985) Tests of Preference and Switching Behavior of the Dragonfly *Celithemis fasciata*. Oikos 44: 350–355.

13. Smout S, Asseburg C, Matthiopoulos J, Fernández C, Redpath S, et al. (2010) The functional response of a generalist predator. PLoS ONE 5: e10761.

**2. References appearing only in Table S2**

Cockrell, BJ (1984a) Effects of temperature and oxygenation on predator-prey overlap and prey choice of *Notonecta glauca.* J Anim Ecol 53: 519-532.

Cockrell, BJ (1984b) Effects of water depth on choice of spatially separated prey by *Notonecta glauca*. Oecologia 62: 256-261.

Cooper, SD, Smith, DW (1982) Competition, predation and the relative abundances of two species of *Daphnia*. J Plankton Res 4: 859-879.

Cothran, ML, Thorp, JH (1985) Tests of prey preference and switching behavior of the dragonfly *Celithemis fasciata*. Oikos 44: 350-355.

Diéguez, MC, Gilbert, JJ (2003) Predation by *Buenoa macrotibialis* (Insecta, Hemiptera) on zooplankton: effect of light on selection and consumption of prey. J Plankton Res 25: 759-769.

Folsom, TC, Collins, NC (1984) The diet and foraging behavior of the larval dragonfly *Anax junius* (Aeschnidae), with an assesment of the role of refuges and prey activity. Oikos 42: 105-113.

Lombardo, P (1997) Predation by *Enallagma* nymphs (Odonata, Zygoptera) under different conditions of spatial heterogeneity. Hydrobiologia 356: 1-9.

Ranta, E, Espo, J (1989) Predation by the rock-pool insects *Arctocorisa carinata, Callicorixa producta* (Het. Corixidae) and *Potamonectes griseostriatus* (Col. Dytiscidae). Ann Zool Fenn 26: 53-60.

Runck, C, Blinn, DW (1994) Role of *Belostoma bakeri* (Heteroptera) in the trophic ecology of a fishless desert spring. Limnol Oceanogr 39: 1800-1812.

Söderström, O, Nilsson, AN (1987) Do nymphs of *Parameletus chelifer* and *P. minor* (Ephemeroptera) reduce mortality from predation by occupying temporary habitats? Oecologia 74: 39-46.

Tate, AW, Hershey, AE (2003) Selective feeding by larval dytiscids (Coleoptera: Dytiscidae) and effects of fish predation on upper littoral zone macroinvertebrate communities of arctic lakes. Hydrobiologia 497: 13-23.

Victor, R, Ugwoke, LI (1987) Preliminary studies on predation by *Sphaerodema nepoides* Fabricius (Heteroptera: Belostomatidae). Hydrobiologia 154: 25-32.
